# Topological robustness analysis of protein interaction networks reveals key targets for overcoming chemotherapy resistance in glioma

**DOI:** 10.1038/srep16830

**Published:** 2015-11-19

**Authors:** Hátylas Azevedo, Carlos Alberto Moreira-Filho

**Affiliations:** 1 Department of Pediatrics, Faculdade de Medicina da Universidade de São Paulo, São Paulo, SP, Brazil

## Abstract

Biological networks display high robustness against random failures but are vulnerable to targeted attacks on central nodes. Thus, network topology analysis represents a powerful tool for investigating network susceptibility against targeted node removal. Here, we built protein interaction networks associated with chemoresistance to temozolomide, an alkylating agent used in glioma therapy, and analyzed their modular structure and robustness against intentional attack. These networks showed functional modules related to DNA repair, immunity, apoptosis, cell stress, proliferation and migration. Subsequently, network vulnerability was assessed by means of centrality-based attacks based on the removal of node fractions in descending orders of degree, betweenness, or the product of degree and betweenness. This analysis revealed that removing nodes with high degree and high betweenness was more effective in altering networks’ robustness parameters, suggesting that their corresponding proteins may be particularly relevant to target temozolomide resistance. In silico data was used for validation and confirmed that central nodes are more relevant for altering proliferation rates in temozolomide-resistant glioma cell lines and for predicting survival in glioma patients. Altogether, these results demonstrate how the analysis of network vulnerability to topological attack facilitates target prioritization for overcoming cancer chemoresistance.

The recognition of state transitions in molecular networks due to environmental or endogenous factors holds the key for elucidating disease mechanisms at the network level^1^. Molecular networks, like gene or protein interaction networks, are usually complex, coordinately regulated and hierarchically organized. Thus, the examination of their topological dynamics after a change of state, such as disease progression or drug resistance, is fundamental for revealing underlying mechanisms and identifying therapeutic targets^2^.

The study of network topology and node hierarchy can be achieved by calculating centrality parameters that determine the importance of each node in a network. The two most commonly used centrality parameters are node degree, which represents the number of direct links a node has, and betweenness, that is the fraction of shortest paths between all pairs of nodes passing through a specific node^3^. The analysis of centrality parameters revealed emergent properties in biological networks, such as their organization into functional modules (also called clusters or communities) and their scale-free topology, i.e. their node degree distribution follows a power-law decay^4^. This last one indicates that most nodes interact with only a few nodes in the network while some nodes exhibit a high number of connections. Highly connected nodes are called hubs and they tend to be essential in protein interaction networks^5^, highlighting the importance of hierarchy for the functioning of molecular pathways. Indeed, the analysis of modules^6^ and topologically relevant nodes^7^ is capable of predicting key regulatory proteins in disease-specific networks.

The topological analysis of scale-free networks demonstrated their high degree of tolerance against network fragmentation after random failures^8^. In contrast, these networks are notably vulnerable to the removal of hubs^8^. Hence, the study of network vulnerability against targeted attack provides an elegant strategy for investigating how these networks are sensitive to the removal of selected nodes representing genes or proteins. An interesting application of this concept lies in cancer drug resistance, considering that cancer cells contain robust biological networks that are resistant to drugs with narrow mechanisms of action^9^. In fact, the study of the topology of molecular networks has already revealed mechanistic insights associated with chemotherapy resistance in cancer^10^,^11^,^12^. This data consequently support a multi-target approach to overcome drug resistance, in which rational therapeutic combinations can be computationally tested in terms of their effects on network parameters.

Particularly for gliomas, primary malignant brain tumors with poor survival rates, the acquired resistance to the alkylating agent temozolomide (TMZ) remains a major challenge limiting its clinical efficacy^13^,^14^. In this field, there is also a paucity of information about the molecular mechanisms underlying TMZ resistance. Thus, we analyzed here the topological features of protein interaction networks linked to TMZ resistance and their resilience against targeted attack in order to reveal key targets for overcoming drug resistance in glioma. These targets were validated *in silico* using proliferation data from temozolomide-resistant glioma cells and co-occurrence relationships between gene expression levels and the prognosis of glioma patients.

## Results

### Network modules participate of biological functions and pathways associated to temozolomide resistance

We utilized network modeling to visualize the interactions between molecules previously associated with glioma resistance to temozolomide (TMZ). With this aim, protein interaction networks were built using molecular information derived from prior studies performed with TMZ-resistant glioma cell lines and human glioma samples. The workflow used for data mining, integration and analysis is described in details in the Material and Methods section.

Table 1 displays the studies used in this work that investigated the molecular basis of TMZ resistance in glioma. Proteins associated with TMZ resistance were compiled (see supplementary Table S1) and utilized as seed nodes to reveal protein interactions and functional modules in the networks. We built two networks, one using information from GeneMania^15^, a Cytoscape plugin that stores protein interactions from different sources, and another using data from the Human Signaling (HS) network^16^, which is a manually curated database containing information of physical interactions between proteins. These two networks are named hereafter GeneMania network and HS network, respectively. The descriptive parameters for the networks are shown in Table 2. Interestingly, these results confirmed the power law distribution (R^2^ = 0.913 and R^2^ = 0.846, respectively) of the node degree values in the TMZ resistance networks, which is concordant with their scale-free topology.

After network construction, groups of densely interconnected nodes (modules) were identified by calculating the clustering coefficient for each node in order to quantify modularity. This parameter estimates the number of links connecting the neighbors of a given node. The more connections a node has with its neighbors, the more its clustering coefficient increases. Then, proteins belonging to each one of these modules were functionally enriched to reveal which biological functions are overrepresented by the clustered nodes.

Using the above approach, we identified functional modules in the GeneMania and HS networks. These subsets of connected proteins may be particularly relevant for TMZ resistance, as proteins belonging to a given module may collectively participate of specific functions. The functional enrichment analysis of these modules showed their involvement in ribosomal functions, cell proliferation and survival, extracellular matrix functions, cell stress, DNA damage response, cell cycle regulation, cell proliferation, migration, growth factor pathways, DNA repair, apoptosis and immune pathways. Moreover, these network modules were associated with pathways related to the growth factors NGF, PDGF and ErbB, interleukins IL-1, IL-2, IL-3, IL-5 and IL-6, as well as to other immunity-related pathways such as MAPK, TGF-β and Toll-like receptors. These modules and their enriched functions are represented in Tables 3 and 4.

### Topological robustness analysis reveals effective strategies for removing nodes in the temozolomide resistance networks

In light of the relationship between modularity and centrality in protein interaction networks, we explored the concept of attacking central nodes in the TMZ resistance networks to interfere with their functional modules. Node degree and betweenness were calculated for all nodes for identifying central nodes in the networks. The product of degree and betweenness (PDB) was also calculated to disclose nodes with both high degree and high betweenness. These measures were used following previous studies where such centrality parameters were assessed for the analysis of attack robustness in complex networks^17^,^18^,^19^. Moreover, while node degree is a purely local centrality measure, betweenness represents a global centrality measure that accounts for the shortest paths among all pairs of nodes. Consequently, using centrality measures from distinct natures is important for analyzing the heterogeneous structure of complex networks^5^.

The above-mentioned centrality values were calculated in the initial networks and used to prioritize the removal of nodes according to different attack strategies. We tested three centrality-based attack strategies for prioritizing node removal. The attack schemes were based on the removal of nodes in the descending order of their centrality values. These strategies were classified as degree-based (nodes with the highest degree were removed first), betweenness-based (nodes with the highest betweenness were targeted first) and PDB-based (nodes with the highest products of degree times betweenness were removed first). We reasoned that top-ranked nodes according to these measures would be essential for maintaining the structure of the networks against targeted attack. Moreover, network robustness was evaluated by assessing how the structure of the networks changed while nodes were being deleted during the removal procedures. The outcome after each round of removal was determined on network robustness by calculating the diameter (d), average shortest path length (a), average inverse path length (1/a), number of nodes or size (S), number of edges (e) and clustering coefficient (cc) in each resulting subgraph. These robustness parameters are commonly applied for studying network behavior during attack simulations^17^,^18^.

**Figure 1:**
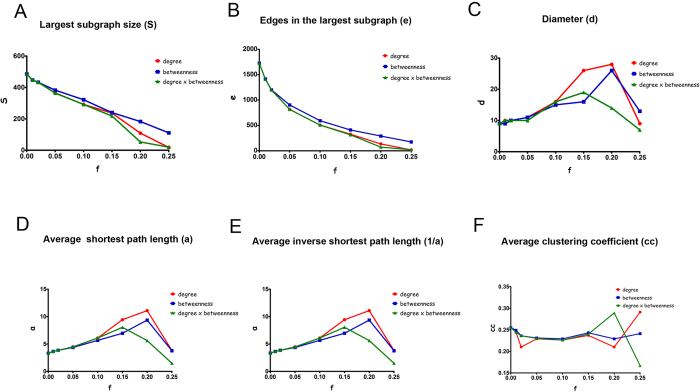
Robustness analysis of the GeneMania network against topological attack. GeneMania network was constructed using information from physical and pathway-related protein interactions contained in GeneMania. Centrality-based attack schemes were focused on the removal of nodes in the descending order of their centrality values. These strategies were classified as degree-based, betweenness-based and product of degree and betweenness-based. The following network robustness parameters were calculated after each round of removal: diameter (**D**), average shortest path length (**A**), average inverse path length (1/a), size (S), number of edges (**E**) and average clustering coefficient (cc) in each resulting subgraph.

**Figure 2:**
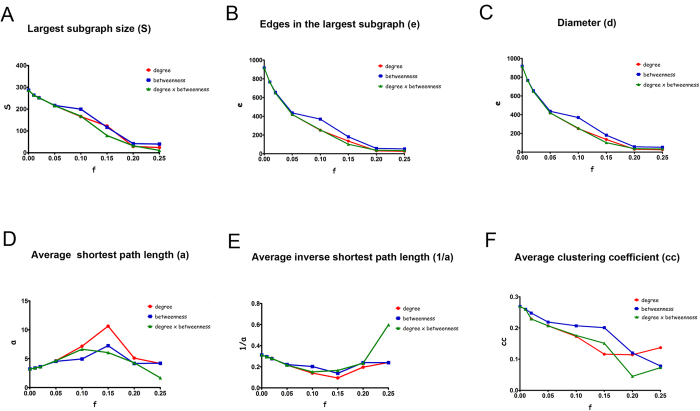
Robustness analysis of the HS network against topological attack. HS Network was constructed using protein interaction information from the Human signaling (HS) network database. Centrality-based attack schemes were focused on the removal of nodes in the descending order of their centrality values. These strategies were classified as degree-based, betweenness-based and product of degree and betweenness-based. The following network robustness parameters were calculated after each round of removal: diameter (**D**), average shortest path length (**A**), average inverse path length (1/a), size (S), number of edges (**E**) and average clustering coefficient (cc) in each resulting subgraph.

Figures 1 and 2 show the comparative performance analysis between the three attack strategies used for removing nodes in the GeneMania and HS networks. For all the removal procedures, the networks were almost totally disconnected after knocking out 20% of the most central nodes, thus indicating their susceptibility to intentional attack. Moreover, by analyzing the graphics in Figs 1A,B and 2A,B, one can conclude that the PDB-based strategy was more effective than the other ones, because the decrease in the largest subgraph size (S) or in the number of edges(e) was more pronounced after the removal of at least 15% of the nodes using this strategy.

The analysis of changes in network diameter (Figs 1C and 2C) and in the average shortest path length (Figs 1D,E and 2D,E) revealed that the degree-based strategy was more effective in decreasing the robustness of the TMZ resistant networks. This occurred because the PDB-based strategy turned the networks smaller in an early stage due to its best performance for those two parameters. Interestingly, although the degree-based strategy reduced the average clustering coefficient after removing 2% of the top central nodes (Figs 1F and 2F), this parameter did not change among the different strategies until 20% of the central nodes were removed. The lowest average clustering coefficient was observed after removing 25% of the central nodes using the PDB-based strategy, corroborating the efficiency of this strategy for interfering with the modularity of the TMZ resistance networks. Interestingly, a higher variation was found in the average clustering coefficient after removing 20–25% of the nodes using the degree- and PDB-based strategies. This observation indicates that broad changes in network modularity precede the decay in network resilience.

### The examination of nodes with both high degree and betweenness identifies known and novel targets for overcoming glioma drug resistance

**Figure 3:**
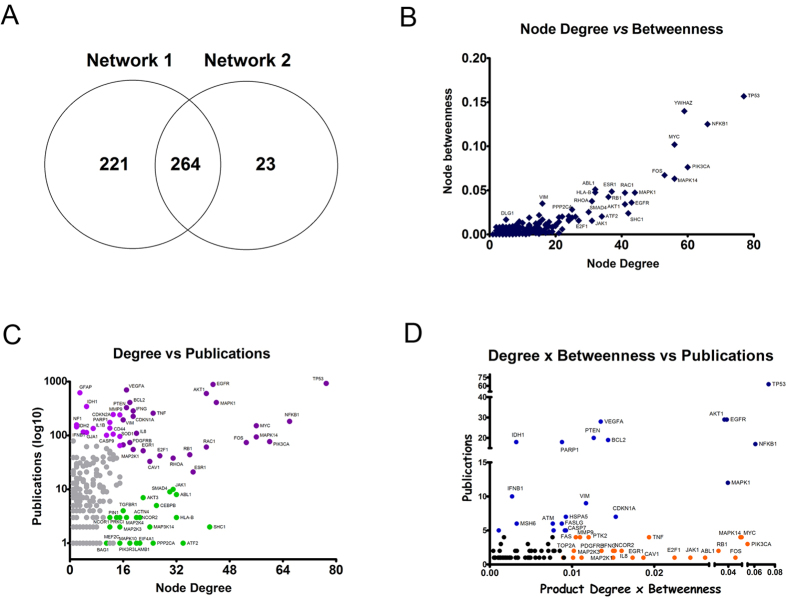
The analysis of nodes with high degree and betweenness identifies known and novel targets for overcoming glioma drug resistance. (**A**) Venn diagram showing that most nodes contained in the HS network are also part of the GeneMania network. (**B**) Scatter plot of degree and betweenness values for all nodes to identify those ones with high relative values in both parameters. (**C**) Scatter plot of node degree values and published studies to identify central nodes less studied in glioma research. Nodes (proteins)less studied in glioma research that also exhibited a high degree were colored in green. Nodes well studied in glioma research were colored in eitherlight or dark purple, according to their low or high degree values, respectively, (**D**) Scatter plot of the product of degree and betweenness values and published studies for each node to identify central nodes less studied in glioma research. Nodes (proteins) less studied in glioma research that exhibited a high degree x betweenness were colored in orange. Nodes well studied in glioma research were colored in either royal or dark blue, according to their low or high degree x betweenness values, respectively,

Considering the above results, we further analyzed the nodes with both high degree and betweenness due to their higher relevance for maintaining the network structure and topological robustness. We decided to center our downstream analysis on the nodes from GeneMania network, considering that most nodes in the HS network were also present in GeneMania network, as displayed in the Venn diagram of Fig. 3A.

We plotted the degree and betweenness values for all nodes in GeneMania network to identify those ones with high values in both parameters (Fig. 3B). A good linear correlation was observed between degree and betweenness values for the nodes (r^2^ = 0.7581), highlighting the hierarchical organization of this network. Additionally, a significant part of these central nodes participate of the PI3K-Akt-mTOR and Ras-Raf-Erk pathways. These pathways were already associated with glioma malignancy and chemoresistance^20^ and thus represent promising targets for drug development in glioma^21^. These central nodes are mainly distributed in the modules 1 and 6 of the TMZ resistance network, corresponding to the biggest clusters identified in the network.

Next, we determined the number of previous studies assessing the relationship between each node and the glioma or temozolomide words, with the aid of GenClip, a literature mining tool. We plotted these values against the degree or against the product of degree and betweenness for each node in order to detect central nodes less studied in glioma research to date (Figures 3C-3D). These nodes were colored in orange in the graph and some examples are Jak1, Pik3ca, Mapk14, Rb1 and E2f1.

### Nodes with high degree and betweenness are important for the viability of temozolomide-resistant glioma cells

We analyzed in silico the potential of nodes with (*i*) high degree (HD), (*ii*) high betweenness (HB) and (*iii*) both high degree and high betweenness (HDB) for altering the viability profile of glioma cells. To achieve this goal, we used the data from the Project Achilles^22^, which contains a database of viability scores across a panel of cancer cell lines after small hairpin RNA (shRNA)-based depletionof target genes. These scores are the log2-based proliferation rates between cells treated with the representative shRNAs and control cells incubated with pooled shRNAs.

**Figure 4:**
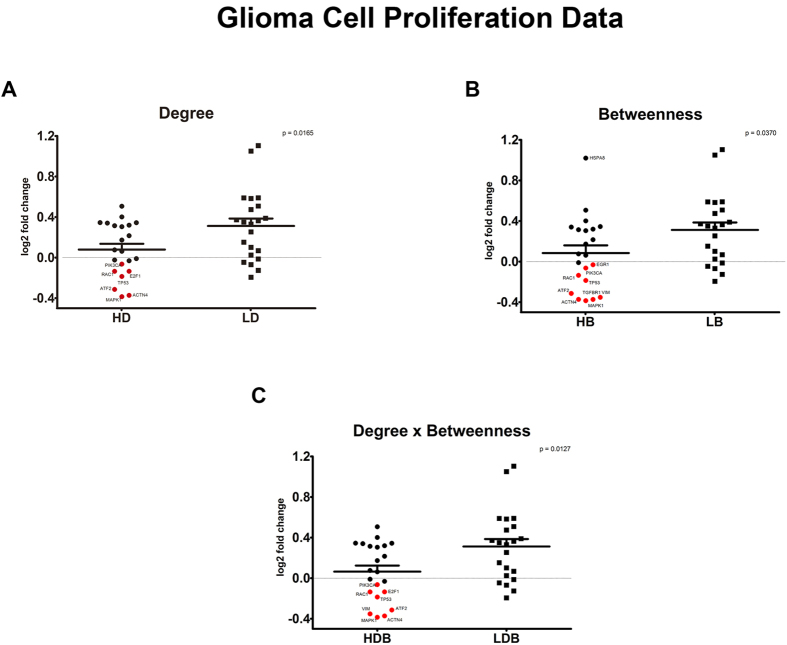
The knock-down of genes high degree and betweenness alter the viability of glioma cells. Comparison between the average log 2 fold changes in cell proliferation after silencing nodes with high or low centralities in temozolomide-resistant glioma cell lines. The viability scores were obtained from the Project Achilles, using information from the cell lines T98, A172, LN382, U21MG and U343. Nodes were divided in high and low degree groups (HD and LD), high and low betweenness (HB and LB) groups or high and low product of degree and betweenness (HDB and LDB) groups. The comparison groups were formed by separating the proteins into two equal groups (**A**–**C**), containing the top or bottom ten percent nodes according to the established centrality rankings. Statistical comparisons were performed between the groups with high and low degree, betweenness or product of degree and betweenness, using Student’s t tests with p < 0.05 as the significance threshold. The high centrality nodes (proteins) that exhibited a prominent change in cell proliferation when silenced were highlighted in red.

The in silico validation was focused on the proliferation data from the glioma cell lines T98G, A172, LN382, U21MG and U343. These cell lines were selected because they are resistant to TMZ treatment, according to previous data^23^,^24^,^25^,^26^,^27^. Interestingly, the comparison of the average log 2 fold changes in cell viability after silencing HD, HB and HDB proteins showed the statistically significant higher pro-death potential of these proteins compared to proteins in the low centrality groups. These results were found when nodes contained in GeneMania network and also analyzed by Project Achilles were separated into two groups: the first one with the top ten percent nodes in terms of centrality values, and the second one with the bottom ten percent nodes. These results are displayed in Fig. 4.

### Proteins corresponding to central nodes in the TMZ resistance network exhibit a higher predictive value for glioma patient survival

**Figure 5:**
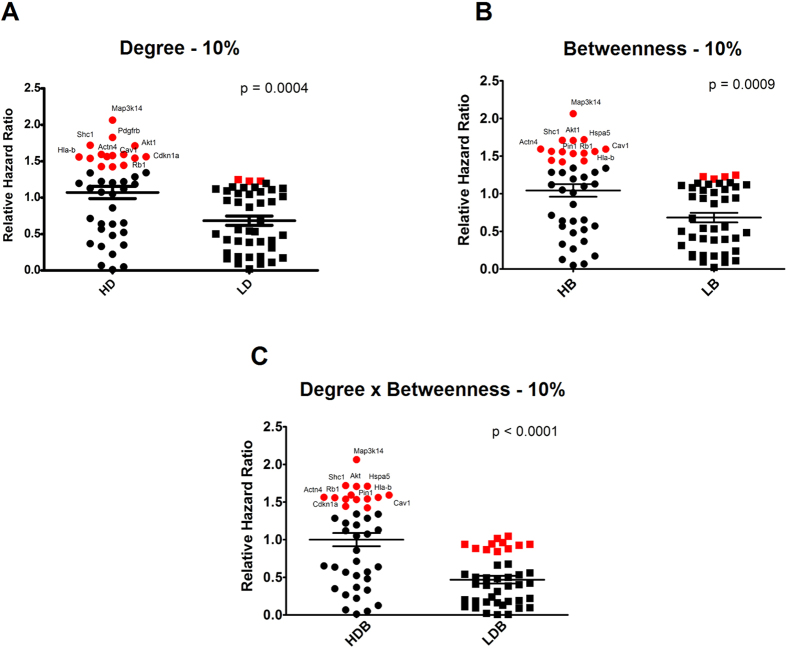
Superior predictive value of proteins with high degree and betweenness for glioma patient survival. Comparison of the survival predictive values between nodes with high and low centralities. The relative hazard ratios were obtained from the Prognoscan database. The relative risk was determined as the relative hazard ratio between groups of patients with high or low expression levels of each protein (ln (HR-high/HR-low). Nodes were divided in high and low degree groups (HD and LD), high and low betweenness (HB and LB) groups or high and low product of degree and betweenness (HDB and LDB) groups. The comparison groups were formed by separating the proteins into two equal groups (**A**–**C**) containing the top or bottom ten percent proteins, according to the established centrality rankings. Statistical comparisons were performed using the absolute relative risks between the groups of nodes with high and low degree, high and low betweenness or high and low product of degree and betweenness, using Student’s t tests with p < 0.05 as the significance threshold. The high centrality nodes (proteins) that exhibited a prominent predictive value for patient death or survival were highlighted in red.

To support the relevance of nodes (i.e. proteins) with high degree and/or betweenness for glioma patients survival, we compared the absolute logarithmic ratio of the relative risks (hazard ratios = HR) between groups of patients with high or low expression levels for each protein (ln (HR-high/HR-low), using data from the PrognoScan database^28^. These results confirmed that central nodes in the TMZ resistance networks are associated with a significant higher risk of patient survival or death when the top and bottom ten percent central nodes were selected for the high and low groups, respectively. These results are summarized in Fig. 5.

## Discussion

The molecular mechanisms of therapeutic resistance have been extensively investigated in cancer research, which resulted in the addition of novel drugs to standard chemotherapies for overcoming cancer resistance^29^. In this context, the analysis of molecular interaction profiles is an interesting approach for prioritizing novel mechanisms of drug combination in cancer^30^. Therefore, we explored here protein-protein interaction networks associated to TMZ resistance in order to predict key targets for tackling glioma drug resistance.

Since cell signaling networks exhibit modular organization, we sought to identify network clusters with specific biological functions that could be involved in glioma resistance. The study of modular organization in cancer signaling networks has already demonstrated that network modularity is correlated with cancer patient survivability^31^. Moreover, proteins belonging to specific hallmarks of cancer^32^ tend to be enriched in particular network modules, and therefore complex processes in cancer cells may involve the participation of several modules. Consequently, the prediction of which hallmarks are predominantly activated in each tumor could be achieved by the identification of major modules obtained from genomics data^33^. Accordingly, we showed here that chemoresistance to TMZ, a DNA alkylating agent, may be attributed not only to a classical hallmark of genome instability, but also to other hallmarks of malignant glioma cells, such as sustained proliferation, cell survival and invasion. Indeed, central nodes in the TMZ resistance network participate of the PI3K-Akt-mTOR and Ras-Raf-Erk pathways, major downstream pathways activated by growth factors during glioma progression^20^,^21^. Moreover, the presence of immune-related modules in the TMZ resistance networks highlights the relevance of immune escape functions for glioma chemotherapy resistance^34^.

We also hypothesized that node hierarchy could influence the efficiency of a candidate target for overcoming chemotherapy resistance^35^. The virtual node knock-out experiments performed here revealed that the PDB-based attack was more effective in interfering with network robustness and modularity, suggesting the TMZ resistance network is susceptible to the attack of nodes with high degree and high betweenness. This is because removing nodes that are hubs (high degree) and bottlenecks (high betweenness) at the same time disconnects groups of nodes in the network, reducing the magnitude of the largest subgraph. The same does not occur when the attack strategy is based on removing nodes that are only hubs, as removing fractions of hubs increases the distance between nodes but they may remain connected in a largest subgraph. Indeed, genes that are defined as hubs and bottlenecks are better correlated with essentiality in cancer networks^36^. These nodes are important because they have a large number of connections and participate at the major intersections between modules in networks^37^. Therefore, the attack of hub-bottlenecks, rather than hub-nonbottlenecks, disclosed the fragility of modular scale-free networks^38^.

We next evaluated *in silico* if knocking-down proteins corresponding to central nodes in the TMZ network could modify the viability of glioma cell lines. The decrease in cell proliferation observed when central nodes are knocked down in temozolomide-resistant cell lines confirms the importance of these nodes for glioma cell viability. Indeed, the association between node centrality and cell viability is observed in different experimental models and nodes (i.e. proteins) that are more important for the survival of an organism are formally called essential^39^. Hence, the integration of molecular interaction profiles and cell viability data may help to validate the extent to which central nodes are essential for cell proliferation in several cancer cell lines.

We also investigated the survival predictive values of nodes displaying high or low centrality values using data from glioma patients. This comparison showed that high centrality nodes have a superior predictive value, confirming that network topological structure is related to cancer patient survival. Indeed, cancer cells exhibiting higher molecular network complexity are more refractory to therapy than those with less complex pathways^40^, corroborating the discovery of novel therapeutic targets through the prioritization of nodes according to their centrality values.

The network topology analysis performed in this study revealed a set of central nodes associated to glioma malignancy, such as Map3k14, Mapk1, Actn4, Hspa5, Atf2, Rac1,E2f1, Shc1, Pik3ca, Akt1, Vim and Egr1. For example, Map3k14, Atf2, Rac1 and Egr1play a role in glioma proliferation and invasion^41^,^42^,^43^,^44^. In parallel, the proteins encoded by Pik3ca, Akt1 and Actn4 are overexpressed in malignant gliomas and correlate with poor survival rates^45^,^46^. Finally, the inhibition of Mapk1 improves the efficacy of temozolomide in brain-implanted tumors^47^ while decreasing E2f1 expression reverses cisplatin resistance in glioblastoma cells^48^. All these proteins may represent interesting targets for testing drug combinations to sensitize glioma cells to temozolomide.

In conclusion, our results show how the topological robustness analysis of protein interaction networks may be helpful for identifying therapeutic targets in cancer. Such approach offers the opportunity to determine target combinations for experimental evaluation by exploring the synergistic effects of targeting multiple proteins on the robustness of drug resistance networks^49^. This analysis showed that investigating nodes with high degree and high betweenness could be an interesting approach for unraveling chemotherapy resistance mechanisms and disclosing novel drug targets. The in silico validation of these findings confirmed the importance of central nodes for tumor cell viability and patient survival in glioma, supporting future studies on network topology features for prioritizing cancer targets.

## Materials and Methods

### Search of genes and proteins related to TMZ resistance in the literature

Data from prior studies performed with TMZ-resistant glioma cell lines and human glioma samples were searched on the database PubMed. We sought for studies describing molecules with functional or expression level association to TMZ resistance in glioma. Functional association was determined when targeting a specific gene or protein led to an alteration in glioma cell viability in the presence of TMZ. Conversely, the expression level association was considered when a TMZ resistance phenotype was correlated with a specific gene/protein differential expression profile. This data was compiled and manually curated in order to use only the most relevant studies in the downstream analysis. Protein names from other species were converted to their human orthologs.

### Construction of the protein interaction networks and identification of functional modules associated with TMZ resistance

The curated dataset was used to build a protein interaction network showing the connections between the molecules related to TMZ resistance. The Cytoscape plugin GeneMania^15^ and the Human signaling network database^16^ were employed to identify physical and pathway interactions between the seed nodes. The descriptive parameters of the networks were calculated using the Cytoscape Plugin Network Analyzer^50^. After the networks were built, groups of densely interconnected nodes named clusters (modules) were identified using the Cytoscape plugin clusterMaker^51^, and the elements belonging to each one of these modules were functionally enriched to reveal overrepresented biological functions, using the online bioinformatics tool Enrichr^52^. The databases KEGG, Wiki pathways, Reactome, Biocarta and Gene Ontology were used for searching relevant enriched functions via Enrichr interface. Only functions with a p-value of less than 0.05 were considered in the analysis.

### Topological Analysis of the TMZ resistance network

Centrality parameters were obtained for all nodes in the networks using the Cytoscape plugin CentiScaPe^53^. The topological parameter degree accounts for the number of edges linked to a given node, while betweenness centrality represents the number of shortest paths passing through a specific node, among the total number of shortest paths in the network.

### Network centrality-based attack strategies

The paradigm between error tolerance and attack vulnerability is usually applied to evaluate the robustness of a scale-free network. In this sense, network topology-based attack simulations were performed here to evaluate the vulnerability of the TMZ resistance network against targeted attack. Nodes were ranked according to their centrality values for prioritizing the node removal of highly scored network nodes. Centrality-based attacks were performed in the original network to determine the impact of orderly removing these nodes on network robustness parameters. The attack strategies were classified as degree-based (nodes with the highest degree were removed first), betweenness-based (nodes with the highest betweenness were targeted first) and product of degree and betweenness-based attacks (nodes with the highest products of degree times betweenness were removed first).

### Robustness analysis of the network against topological attack

The robustness of the networks associated with TMZ resistance was determined by calculating some robustness parameters after rounds of removal based on deleting fractions of five percent of the nodes at each removal time. The robustness parameters evaluated were: i) diameter; ii) size of the largest connected subgraph; iii) number of edges in the largest connected subgraph; iv) average geodesic path; and v) average inverse geodesic path. The calculation of these parameters was implemented using the Cytoscape Plugin Network Analyzer^50^ and only the largest subgraph in each round of removal was considered to calculate the above parameters.

### Relationship between node centrality and novelty in glioma research

The degree and betweenness value for all nodes were plotted in scatter plots to identify nodes with high values in both centrality parameters. In addition, these scatter plots were constructed using either the degree or the product of degree and betweenness values (x coordinate) against the number of studies previously investigating the relationship between each node and glioma or temozolomide words (y coordinate), with the aid of the literature mining tool GenClip^54^. The scatter plots were built using the GraphPad Prism® 5.

### Examination of the importance of central nodes for the proliferation of TMZ-resistant glioma cells

The effects on cell viability of knocking down specific proteins were evaluated employing data from Project Achilles, using information from the temozolomide-resistant glioma cell lines T98, A172, LN382, U21MG and U343. The average log2-fold change in cell proliferation was calculated for each gene, using data from the corresponding cell lines. The differing role of central nodesfor the proliferative state of TMZ-resistant glioma cells was determined by comparing groups with high and low centrality values. The groups were formed by selecting the top and bottom ten percent proteins in terms of their centrality values. These ten percent highest and ten percent lowest-ranked proteins were identified in the initial network based on their degree, betweenness or product of degree and betweenness.

### Analysis of the predictive value of central nodes for glioma patients’ survival

Analogously to the analysis performed with the cell proliferation data from Project Acchiles, glioma patient survival data (Prognoscan database) was used to determine if higher centrality nodes would have a superior predictive value than lower centrality nodes in the TMZ resistance network. The absolute relative risks (hazard ratios = HR) between groups of patients with high or low expression levels for each protein (ln (HR-high/HR-low) were obtained for each protein and only significant results (adjusted p value < 0.05) were used in the final analysis. The distinct predictive value of central proteins was assessed by comparing groups with high and low centrality nodes. These groups were formed by selecting the top and bottom ten percent proteins in terms of centrality values. These ten percent highest and ten percent lowest-ranked proteinswere identified in the initial network based on their degree, betweenness or product of degree and betweenness.

### Statistical analyses between the high and low centrality groups

The statistical comparisons between the groups with high and low degree, betweenness or product of degree and betweenness were made using Student’s t tests with p < 0.05 as the significance threshold, using the GraphPad Prism® 5.

## Additional Information

**How to cite this article**: Azevedo, H. and Moreira-Filho, C. A. Topological robustness analysis of protein interaction networks reveals key targets for overcoming chemotherapy resistance in glioma. *Sci. Rep.* **5**, 16830; doi: 10.1038/srep16830 (2015).

## Supplementary Material

Supplementary Information

## Figures and Tables

**Table 1:** Selected published studies assessing molecules linked to temozolomide resistance in gliomas.

Article	Title	Association	Reference
Huang *et al.*, 2014	The microarray gene profiling analysis of glioblastoma cancer cells reveals genes affected by FAK inhibitor Y15 and combination of Y15 and temozolomide	Expression level	55
Epple *et al.*, 2013	Induction of the unfolded protein response drives enhanced metabolism and chemoresistance in glioma cells	Expression level	56
Bruyère *et al.*, 2011	Temozolomide-induced modification of the CXC chemokine network in experimental gliomas	Expression level	57
Cui *et al.*, 2010	Decoupling of DNA damage response signaling from DNA damages underlies temozolomide resistance in glioblastoma cells.	Functional	58
Yoshino *et al.*, 2010	Gene expression profiling predicts response to temozolomide in malignant gliomas.	Expression level	59
Auger *et al.*, 2006	Genetic alterations associated with acquired temozolomide resistance in SNB-19, a human glioma cell line	Expression level	60
Demuth *et al.*, 2007	MAP-ing glioma invasion: mitogen-activated protein kinase 3 and p38 drive glioma invasion and progression and predict patient survival	Functional	61
Ye *et al.*, 2013	Protective properties of radio-chemoresistant glioblastoma stem cell clones are associated with metabolic adaptation to reduced glucose dependence.	Expression level	62
Kumar *et al.*, 2013	Temozolomide-modulated glioma proteome: role of interleukin-1 receptor-associated kinase-4 (IRAK4) in chemosensitivity.	Expression level	63
Happold *et al.*, 2012	Distinct molecular mechanisms of acquired resistance to temozolomide in glioblastoma cells	Expression level	64
Zhang *et al.*, 2010	Acquired resistance to temozolomide in glioma cell lines: molecular mechanisms and potential translational applications.	Functional	65
Gimenez *et al.*, 2012	Quantitative proteomic analysis and functional studies reveal that nucleophosmin is involved in cell death in glioblastoma cell line transfected with siRNA.	Expression level	66

**Table 2:** Descriptive parameters for the molecular networks associated with temozolomide resistance in glioma.

Network parameters	GeneMania Network	HS Network
Number of nodes	485	287
Number of edges in the largest subgraph (e)	1725	917
Number of shortest paths	234,740	82,082
Largest connected subgraph size (S)	485	287
Diameter (d)	9	7
Average number of neighbors	7.113	6.390
Network density	0.015	0.022
Average shortest path length (a)	3.321	3.213
Network heterogeneity	1.380	1.376
Clustering coefficient	0.255	0.269
Network centralization	0.145	0.192
R^2^ value for power-law curve fitting of node degree distribution	0.913	0.846

**Table 3:** Main functions and pathways enriched in each moduleofthe GeneMania network.

Module	Number of proteins	Main enriched functions and pathways
1	97	MAPK signaling pathway, toll-like receptor cascade, autophagy, cellular senescence
2	84	apoptosis, DNA damage response, cytoplasmic ribosomal proteins, ATM signaling pathway, regulation of cell cycle progression, p53 pathway
3	94	amino acid metabolism, unfolded protein response, antigen processing and presentation, ubiquitin-proteasome system
4	17	Mitotic G1-G2/M phases, centrosome maturation
5	8	detoxification of reactive oxygen species, cellular response to stress
6	112	focal adhesion, regulation of actin cytoskeleton, ECM-receptor interaction, cytokine-cytokine receptor interaction, NGF, PDGF, SCF-Kit, DAP12 and PI3K/Akt pathways.
7	16	apoptosis
8	19	matrix metalloproteinases, Insulin-like growth factor uptake
9	15	TGF-β receptor signaling pathway
10	9	cell cycle and mRNA processing

**Table 4:** Main functions and pathways enriched in each module of the HS network.

Module	Number of proteins	Main enriched functions and pathways
1	86	Cytokine-cytokine receptor interaction, melanoma, JAK-STAT signaling pathway, MAPK signaling pathway, TLR signaling pathway, apoptosis, glioma, ESC Pluripotency Pathways, IL-1 pathway, senescence and autophagy, signaling by ERBB4, SCF-KIT, ERBB2, FGFR andPDGF
2	12	mRNA processing, Mitotic anaphase, negative regulation of the PI3K/AKT network
3	13	Tight junction, calcium signaling pathway, signaling pathways in glioblastoma, endothelin pathway, DNA damage response, calcitonin-like ligand receptors, negative regulation of the PI3K/AKT network
4	33	Regulation of action cytoskeleton, adherens junctions, cell cycle, ATM signaling pathway, TP53 signaling pathway, DNA damage response, EPH-Ephrin signaling
5	55	ECM receptor interaction, focal adhesion, cell communication, WNT signaling pathway, IL6 and IL3 pathways, TGF-beta signaling pathway, negative regulation of MAPK cascade
6	15	Ribosome, NOD pathway, translation factors, caspase cascade in apoptosis
7	54	MAPK signaling pathway, apoptosis, cellular response to stress, toll-like receptor cascade,
8	17	Notch signaling pathway, calcium signaling pathway, insulin signaling pathway
